# Vitamin D Deficiency as It Relates to Oral Immunity and Chronic Periodontitis

**DOI:** 10.1155/2018/7315797

**Published:** 2018-10-01

**Authors:** R. A. G. Khammissa, R. Ballyram, Y. Jadwat, J. Fourie, J. Lemmer, L. Feller

**Affiliations:** Department of Periodontology and Oral Medicine, Sefako Makgatho Health Sciences University, Medunsa 0204, South Africa

## Abstract

The biologically active form of vitamin D, 1,25 dihydroxyvitamin D (1,25(OH)_2_D) and its receptor, the vitamin D receptor (VDR), play roles in maintaining oral immunity and the integrity of the periodontium. Results of observational cross-sectional clinical studies investigating the association between vitamin D serum level and the incidence and severity of chronic periodontitis indicate that, perhaps owing to the immunomodulatory, anti-inflammatory, and antibacterial properties of 1,25(OH)_2_ D/VDR signalling, a sufficient serum level of vitamin D is necessary for the maintenance of periodontal health. In cases of established chronic periodontitis, vitamin D supplementation is associated with reduction in the severity of periodontitis. As cross-sectional studies provide only weak evidence for any causal association and therefore are of questionable value, either longitudinal cohort studies, case controlled studies, or randomized control trials are needed to determine whether or not deficiency of vitamin D is a risk factor for chronic periodontitis, and whether or not vitamin D supplementation adjunctive to standard periodontal treatment is in any way beneficial. In this article, we discuss the relationship between vitamin D, oral immunity and periodontal disease and review the rationale for using vitamin D supplementation to help maintain periodontal health and as an adjunct to standard periodontal treatment.

## 1. Introduction

Chronic periodontitis is an inflammatory disease caused by dentogingival bacterial plaques and if left untreated, it causes progressive destruction of periodontal tissues, ultimately leading to tooth loss. In a subset of subjects with chronic periodontitis, there may be an increased risk of cardiovascular disease, diabetes mellitus, and complications of pregnancy [1–4]. Periodontitis affects up to 50% of the adult population [5–7].

**Figure 1:**
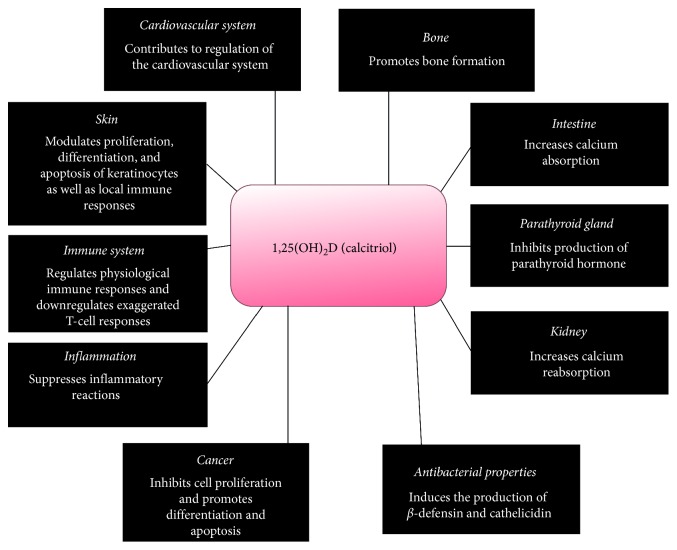
The functions of vitamin D.

Vitamin D plays a role in maintaining the homeostasis of various biological systems including the neuromuscular, skeletal, cutaneous, cardiovascular, and immune systems. In addition, vitamin D has tumour suppressing, anti-inflammatory, and antibacterial properties [8–12] (Figure 1). While there is no doubt about the essential role of vitamin D in maintaining bone and calcium homeostasis, its role in other biological systems is less well-defined [13].

Cross-sectional observational studies show that vitamin D deficiency may be associated with increased risk of chronic periodontitis [1, 6, 14–17], and that supplementation with vitamin D alone, or with vitamin D together with calcium may help to maintain periodontal health, may increase mineral density of the jaws, and may inhibit inflammatory alveolar bone resorption [15, 18–21]. Furthermore, in subjects with adequate vitamin D, surgical treatment for chronic periodontitis appears to be more successful than in subjects with vitamin D deficiency [22]. However, results of some longitudinal studies show that vitamin D deficiency is a poor predictor of progressive tissue destruction in subjects with chronic periodontitis [7] and conversely, vitamin D sufficiency does not protect against progression of chronic periodontitis [23]. These longitudinal studies do not provide any information regarding the association between vitamin D levels and chronic periodontitis in the general population. The two studies deal only with a selected population of men over the age of 65 recruited for a study of “Osteoporotic Fractures in Men” [7], and with a selected population of postmenopausal women enrolled in the “Buffalo OsteoPerio Study” [23].

Cross-sectional observational studies also show that vitamin D deficiency, independently of chronic periodontitis, is associated with increased risk of cardiovascular disease [9, 10, 24], but it is not known if concurrence of periodontal disease and vitamin D deficiency poses a cumulative cardiovascular risk. In any case, there are no strong evidence-based data to show that supplementation with vitamin D reduces the incidence or the severity of cardiovascular or of any immunoinflammatory diseases [9, 24, 25]. Nevertheless, vitamin D has been used in prevention or treatment of a number of infections including respiratory infections [26], gingivitis [27], and influenza [28] and in the management of asthma [29]. Owing to climatic variations and variations in skin pigmentation, according to generally accepted norms of serum levels of vitamin D, deficiency of vitamin D is very common. In fact, if vitamin D deficiency is defined as a serum level of 25(OH)D below 50 nmol/L, then up to 40% of Europeans can be considered to be deficient [30].

Standard treatment of periodontal disease focuses on reducing the dentogingival bacterial load through personal and professional mechanical disruption of the biofilm, by the use of local or systemic antibacterial agents, or by downregulating the immunoinflammatory response with drugs in order to reduce the bacteria-induced inflammation and to arrest the progression of periodontal tissue damage [31]. If indeed vitamin D were to be found to be effective in the prevention and treatment of periodontitis, then it should be added to the arsenal of biologically active therapeutic agents.

In this narrative review, we describe the possible mechanisms by which vitamin D deficiency may play roles in the pathogenesis of chronic periodontitis and in maintaining the homeostasis of the oral epithelium and the integrity of oral immunity.

## 2. Vitamin D and Vitamin D Receptor (VDR)

About 80% of vitamin D in the body is derived from ultraviolet B (UVB)-induced photoconversion in the skin of 7-dehydrocholesterol to vitamin D_3_ (cholecalciferol) and the remainder from animal dietary sources in the form of vitamin D_3_ or of vitamin D_2_ (ergocalciferol). In a modern diet, food supplements substantially augment the natural sources. The term vitamin D refers either to vitamin D_2_ or to vitamin D_3_ or to both, and either can be used for correcting vitamin D deficiency [25, 32].

Exposure to sunlight is essential for achieving a sufficient level of vitamin D [25, 29, 33], but as the available evidence suggests that excessive exposure to sunlight raises the risk of skin cancer, it is common practice to avoid exposure to sunlight or to wear protective clothing and to use sunscreen with high protection factors when outdoors [34, 35]. Under these circumstances, it is difficult without supplementation to attain sufficient levels of vitamin D for vitamin D-related physiological activities [25]. Because melanin reduces the penetration of UVB into the skin, diminishing the photoproduction of vitamin D, black people are more frequently vitamin D deficient than white people [5, 30]. This may contribute minimally to the greater severity of chronic periodontitis in blacks than in whites [5].

**Figure 2:**
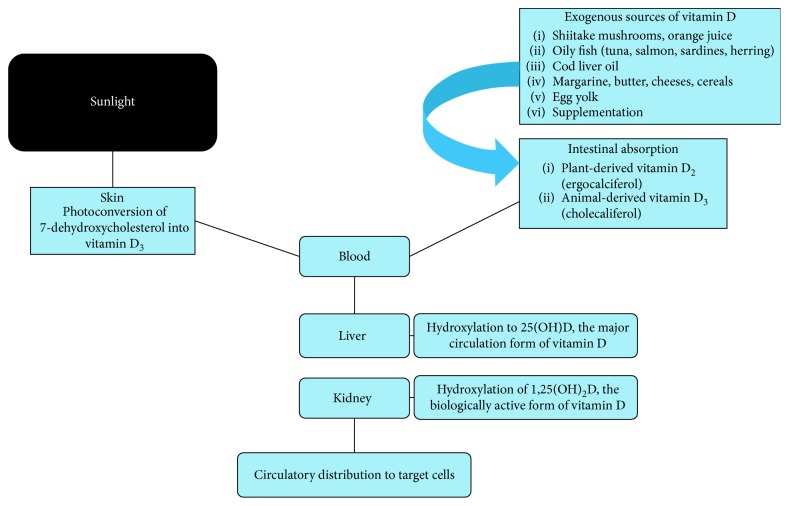
Synthesis of vitamin D precursors and metabolites.

Both vitamin D_2_ and D_3_ are biologically inactive and are converted in the liver into 25(OH)D which then is converted mainly by the proximal tubular cells of the renal nephrons into 1,25(OH)_2_D. This is the biologically active vitamin D (Figure 2). However, other tissues can also to a lesser extent convert vitamin D2 and D3 to the biologically active form [13, 25, 32, 36].

As the 1,25(OH)_2_D has a half-life of only about 4 hours, 25(OH)D with a half-life of 2-3 weeks is used to determine serum levels of vitamin D. Metabolites of vitamin D are transported in the circulation by the vitamin D binding protein, and upon reaching their target cells, they dissociate from the binding protein and enter the cells [8, 19, 25, 32, 37, 38]. The biochemical properties of vitamin binding protein which is the principle transporter of vitamin D and its metabolites, determine the free levels of free vitamin D available to the tissues [33].

Regulation of the concentration of circulating 25(OH)D and 1,25(OH)_2_D is a complex process which is modulated by multiple factors including age, sunlight exposure (duration and intensity), diet (oily fish such as tuna, salmon, sardines; cod liver oil; yeast and fungi), plasma calcium, parathyroid hormone, direct feedback by 1,25(OH)_2_D, fibroblast growth factor 23, diseases (i.e., malabsorption syndromes, sarcoidosis, and impaired calcium metabolism), systemic inflammatory reactions, and medications (i.e., glucocorticoids, anticonvulsants, and barbiturates) [13, 25, 36, 38, 39]. As older adults are often less exposed to sunlight and have reduced capacity to produce biologically active vitamin D metabolites and to absorb vitamin D from the intestine and may be suffering from chronic diseases requiring multiple-drug treatment, they are at particular risk of vitamin D deficiency [12].

According to the Endocrine Society Clinical Practice Guideline [25], Vitamin D deficiency is defined as levels of 25(OH)D below 50 nmol/L and insufficiency as 25(OH)D levels of 52.5–72.5 nmol/L. For people with vitamin D deficiency, in order to attain blood levels of 25(OH)D above 75 nmol/L, they should be treated daily with 6000 IU of either vitamin D_2_ or vitamin D_3_, followed by a maintenance dose of 1500–2000 IU/day [25, 29]. Although about 70 nmol/L of vitamin D enhances both calcium and phosphorus absorption from the intestine and enhances bone health and muscle function, the adequate levels of 25(OH)D for nonskeletal tissue health are unknown [25]. For the best clinical outcomes of vitamin D supplementation, daily doses are better than higher weekly or monthly doses [12] because daily doses result in a more stable serum and tissue concentration [38].

1,25(OH)_2_D exerts most of its activities through the widespread vitamin D nuclear receptor (VDR) which functions as a transcription factor. VDR forms a heterodimer with the retinoid X receptor (RXR), and this VDR/RXR binds to vitamin D response elements in target genes, regulating gene expression either by activation or by repression of gene transcription [8, 10, 40].

1,25(OH)_2_D/VDR/RXR-induced transcription of target genes is modulated by other transcriptional coactivators and corepressors which are recruited to the vitamin D response elements. However, VDR may mediate cellular functions in a ligand-independent manner [13]. 1,25(OH)_2_D/VDR and glucocorticoid receptor intracellular signalling pathways cross-talk so that increased levels of vitamin D may upregulate responsiveness of certain target cells to glucocorticoids; and as VDR and glucocorticoid receptor share some transcriptional coactivators, VDR may promote transcription of certain genes induced by glucocorticoids [13].

The 1,25(OH)_2_D/VDR signalling pathway interacts with other signalling pathways in the regulation of many biological processes, including calcium and bone homeostasis, inflammation, cell mediated immunity, cell-cycle progression, and apoptosis [8]. The 1,25(OH)_2_D/VDR signalling pathway has the capacity to mediate antibacterial, antiviral, and anti-inflammatory activity [29]. VDR polymorphism has been associated with increased risk of several diseases, with some of the genetic variants being less responsive than others to 1,25(OH)_2_D in suppressing inflammatory processes, thus favouring the development of cutaneous inflammatory conditions [41] and possibly of chronic periodontitis [5, 42].

1,25(OH)_2_D/VDR signalling in osteoblasts may cross-talk with the transforming growth factor *β*, insulin growth factor 1, interferon, parathyroid hormone, and Wnt/*β* catenin signalling pathways to mediate physiological activities of osteoblasts [13]. 1,25(OH)_2_D/VDR pathways directly or indirectly can mediate differentiation and maturation of osteoblasts and osteoclasts, thus influencing bone remodelling. 1,25(OH)_2_D/VDR pathways in osteoblasts enhance the expression of osteogenic genes such as those encoding type I collagen, alkaline phosphatase, osteocalcin, and osteopontin which drive bone formation and upregulate the expression by osteoblasts of RANKL which subsequently promotes differentiation and activity of osteoclasts [13]. Vitamin D deficiency has the potential to interfere with bone homeostasis, but as long as calcium serum levels are normal, bone metabolism appears not to be affected by vitamin D deficiency [13].

## 3. Oral Mucosal Immunity

The oral mucosal epithelium separates a microorganism-ridden environment from the underlying connective tissue. It acts as the physical barrier that protects the deeper tissues from penetration of water and a wide range of water-soluble molecules, from invasion by microorganisms with their associated antigens and toxins, and from minor mechanical damage [43]. 1,25(OH)_2_D is produced, and VDR is expressed by keratinocytes of the basal and spinous layers of the oral epithelium, and 1,25(OH)_2_D/VDR signalling influences proliferation, differentiation, and apoptosis of keratinocytes, and local immune responses [8, 44]. In fact, 1,25(OH)_2_D/VDR signalling in oral keratinocytes mediates antiproliferative and prodifferentiation effects, and vitamin D-deficient laboratory animals show increased proliferation of oral epithelium without any morphological or histological abnormalities [45].

The epithelium and the underlying lamina propria of the oral mucosa are populated by innate immune cells including macrophages, natural killer (NK) cells, NKT cells, polymorphonuclear leukocytes, and dedicated antigen-presenting cells, with all their related cytokines and chemokines [43]. In response to antigenic stimulation, activated keratinocytes produce antimicrobial agents such *β*-defensins and cathelicidins and can mediate immunoinflammatory reactions [46]. Salivary flow, salivary secretory immunoglobulin A, and gingival crevicular fluid are additional physical and biological elements of oral mucosal immunity [43].

Oral mucosal immunity has many functions including control of colonization of the oral mucosa by pathogenic microorganisms, generation of protective immunoinflammatory responses against invading pathogens, mediation of immune tolerance to commensal microorganisms and foreign antigens derived from exogenous sources, and neutralization of harmful exogenous antigens [47].

Oral keratinocytes and innate immune cells in the lamina propria of the oral mucosa express molecular pattern-recognition receptors that can detect microorganisms and harmful endogenous molecules derived from tissue damage. There are several families of molecular pattern-recognition receptors including the Toll-like receptor (TLR) family, the c-type lectin receptor family, and the mannose receptor family [46, 47]. Stimulation of TLR receptors by periodontopathic bacteria breaching the crevicular epithelium triggers the production of antibacterial and chemotactic agents, inflammatory mediators, and cytokines. All of these induce a nonspecific inflammatory reaction and mobilize dedicated antigen-presenting cells to the infected gingival site. In turn, these biological reactions initiate and drive adaptive immunoinflammatory reactions [43, 46, 47].

Invasion of the gingival epithelium by periodontopathic bacteria brings about activation of keratinocytes, myeloid dendritic cells, and macrophages. After having recognized molecular patterns of periodontopathic bacteria through TLRs, local immature myeloid dendritic cells process the pathogenic antigen and undergo maturation, with upregulation of expression of major histocompatibility complex (MHC) and costimulatory surface molecules. In the presence of MHC surface molecules, the mature dendritic cells can effectively present antigens to the naïve T cells in draining lymph nodes, initiating cross-priming and mediating the generation of T cell immune responses [43].

The primed T cells in the lymph nodes then differentiate into antigen-specific memory effector CD4+ and CD8+ T cells and into regulatory T (Treg) cells. The subtype and the magnitude of the antigen-specific T cell response in the lymph nodes is determined by the nature of the infective agent, by the cytokine profile in the microenvironment, by the specific T cell receptor repertoire, and by the profile of the cell surface molecules expressed by antigen-presenting dendritic cells [43, 47]. Some of the effector T cells will remain in the lymph nodes, others will enter the circulation, and those which reach the oral mucosa will engage in local effector immune responses and in immune surveillance [43].

In the lymph nodes, IL-12 and IL-18 will generate a Th1 immune response mediated by IL-2, INF-ϒ, and TNF; IL-4 will generate a Th2 immune response mediated by IL-4, IL-5, IL-6, and IL-13; and TNF-*β*, IL-1*β*, and IL-6 will generate a Th17 immune response mediated by IL-17, IL-21, and IL-22 [46]. In addition, Treg cells *via* IL-10 downregulate the induction of T-cell-mediated immune responses in the lymph nodes and suppress the activity of T-cells in the peripheral tissue, thus mediating immune tolerance and preventing upregulation of immunoinflammatory reactions [43, 47]. Despite this process of T cell polarization, the polarized T cells retain some functional versatility, having the capacity to produce cytokines which are not considered lineage-specific [43].

In the context of immune homeostasis, the 1,25(OH)_2_D/VDR signalling pathway can modulate the production of the proinflammatory cytokines IL-2, IL-17, and INF-*α*; suppress the maturation of antigen-presenting dendritic cells with the consequent decrease in antigen-specific T cell activation and proliferation; and promote the activity of Treg cells. Together, these fine-tuned physiological immune responses can downregulate hyperactive T cell mediated immunoinflammatory reactions and moderate autoimmune T cell responses [9, 11, 48–50].

However, despite all this, the evidence for a causal association between vitamin D deficiency and the incidence and severity of immunoinflammatory diseases is weak, and augmenting standard treatment with vitamin D supplementation or its biologically active analogues does not seem to improve the efficacy of the treatment of any autoimmune or immunoinflammatory diseases [9, 51].

In addition, it has been shown that VDR polymorphism, in the presence of exogenous aetiological factors (i.e., tobacco smoke and alcohol), is associated with increased risk of chronic periodontitis and other inflammatory conditions. Some genetic variants may be less responsive to 1,25(OH)_2_D in suppressing inflammation, thus favouring bacteria-induced tissue damage, while other variants are associated with low bone-mineral density, thus making alveolar bone vulnerable to bacterial plaque-induced inflammatory bone resorption [5, 41, 42, 52, 53].

## 4. Periodontitis

**Figure 3:**
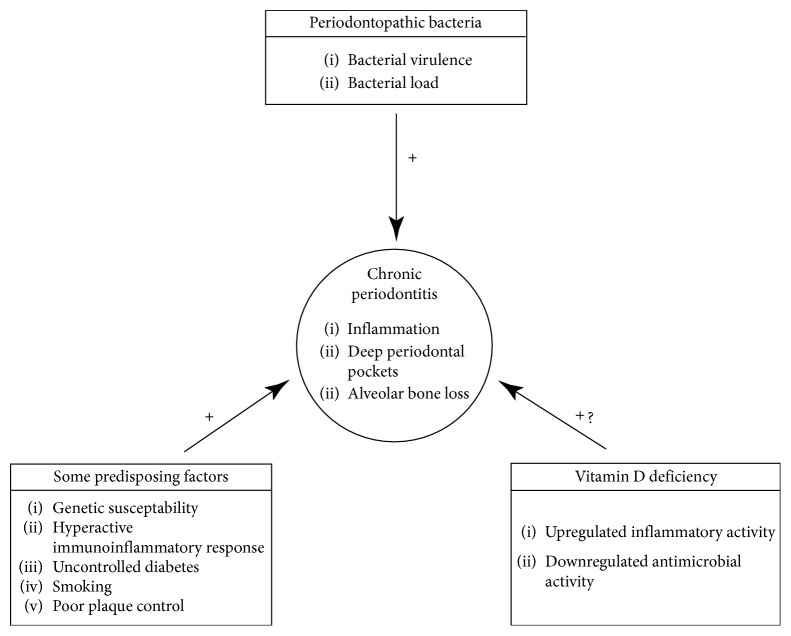
Pathogenesis of periodontal disease.

Periodontitis is a bacterial plaque-induced inflammatory disease characterized by exudation of cervicular fluid, increased periodontal probing depths, bleeding on probing, and loss of alveolar crestal bone. The pathogenesis is multifactorial with complex interaction between bacterial agents and bacteria-induced immunoinflammatory responses, on a background of inherent genetic predisposition. Risk factors such as smoking, uncontrolled diabetes, vitamin D deficiency, and deep periodontal pockets that favour proliferation of periodontopathic bacteria, all have the capacity to aggravate the course of the disease (Figure 3) [52–54].

The metabolic products of early, mainly aerobic bacterial colonisers, together with environmental organic and inorganic compounds form a biofilm in which the pioneer bacteria multiply. The chemical and physical properties of this biofilm within the ecological niche favour proliferation of late bacterial colonisers, including anaerobic periodontopathic bacteria. The biofilm is retained *in-situ* by its adhesive and cohesive properties and provides some protection to the bacterial flora against penetration of antibiotics [54].

In gingival health, the commensal bacteria including Gram-positive facultative cocci and rods and some anaerobes and the gingival tissues are in biological equilibrium. Alterations in the local microenvironment or in the host's immunity may favour multiplication of Gram-negative anaerobic bacterial species, including the periodontopathic bacteria *Porphyromonas gingivalis*, *Aggregatibacter actinomycetemcomitans*, *Prevotella intermedia*, and *Treponema denticolo*, bringing about a disturbance in the host-bacterial biological equilibrium [54].

*P. gingivalis*, the main periodontopathic bacterium, possesses a number of virulence factors and micromorphological structures including gingipain, lipopolysaccharides (LPS), fimbriae, and outer membrane vesicles which individually or together can cause direct tissue damage [55]. After having become attached to a gingival sulcular epithelial cell, *P. gingivalis* enters the cell inducing remodelling of the actin and tubulin cytoskeleton, and after intracellular multiplication, it spreads *via* actin bridges to neighbouring cells, thus infecting a field of epithelial cells. Within the infected cell, *P. gingivalis* can survive, ultimately inducing apoptosis, lysis of the infected cell [31, 55, 56], and thence invading connective tissue cells and osteoblasts of the alveolar bone. *P. gingivalis* can then inhibit differentiation and ultimately induce apoptosis of infected osteoblasts, thus inhibiting bone turnover [57, 58].

In subjects with chronic periodontitis, the expression of TLR2 and TLR4 by cells of the periodontium is upregulated in response to the preponderance of periodontopathic bacteria [55]. Lipopolysaccharides of *P. gingivalis*, *via* TLR 2/4, trigger the activation of the transcription factors NF-*κ*B, AP-1 (activator protein 1), and the STAT-3 (nuclear signal transducers and activators of transcription-3). These upregulate the expression of genes encoding inflammatory mediators including cytokines, chemokines, prostaglandins, and proteinases [31, 55, 59], generating an initial inflammatory reaction. This reaction will be amplified and propagated by activated immunoinflammatory cells of the adaptive arm of the immune system recruited to the infected periodontal site [31], causing marginal alveolar bone loss with increased periodontal pocket depths, raised pH, and decreased local redox potential. All these together favour proliferation of anaerobic bacteria, and unless interrupted by treatment, a vicious cycle of bacterial multiplication, inflammation, and progressive alveolar bone destruction will occur, ultimately resulting in tooth loss [54]. Thus, while bacteria do cause some direct tissue damage, most of the damage of chronic periodontitis is mediated by immunoinflammatory reactions in response to the challenge of the periodontopathic bacteria [59].

The interaction between molecular patterns of certain periodontopathic bacteria and TLR1/2 of innate oral immunocytes including monocytes/macrophages and keratinocytes may also induce the expression of VDR and the production of 1,25(OH)_2_D by these cells. In turn, the 1,25(OH)_2_D/VDR signalling induces the expression of genes encoding the antibacterial agents cathelicidin and *β* defencin [1, 10, 51] which may provide some protection against the development of bacterial plaque-induced chronic periodontitis [13].

Alveolar bone loss in chronic periodontitis is brought about by increased expression of receptor activator of nuclear factor kappa-Β ligand (RANKL), by Th17-derived IL-17 and by TNF-*α* all of which have the capacity directly or indirectly to promote osteoclastogenesis. Lipopolysaccharides of anaerobic bacteria, *via* stimulation of TLRs, upregulate the expression of RANKL by fibroblasts, osteoblasts, and/or by T and B lymphocytes, resulting in differentiation and activation of osteoclasts; Th17-derived IL-17 can also upregulate the expression of RANKL by osteoblasts and CD4+ T cells; and TNF-*α* produced by neutrophils, macrophages, and Th1 cells directly promotes osteoclastogenesis. All these bring about the resorption of supporting alveolar bone characteristic of chronic periodontitis [31].

In progressive chronic periodontitis, dissemination of periodontopathic bacteria and of inflammatory mediators from the inflamed periodontal tissues is not uncommon. As a result, in a subset of genetically predisposed persons with upregulated immunoinflammatory responses, active chronic periodontitis can increase the risk of cardiovascular disease, stroke, inadequate glycaemic control in diabetes mellitus, and complications of pregnancy [1, 4]. Indeed, it has been reported that after adjustment for confounding variables, compared to those with minimal periodontal disease, subjects with advanced periodontitis have a 25% increased risk of coronary heart disease [4].

The statistical association between vitamin D status represented by 25(OH)D levels and resistance to periodontal disease observed in cross-sectional studies might be explained in terms of several biological mechanisms. Firstly, 1,25(OH)_2_D, the biologically active form of vitamin D, through its positive role in maintaining calcium and bone homeostasis can increase the mineral density of alveolar bone, and thus may reduce alveolar bone resorption, with a consequent decrease in the severity of chronic periodontitis and may help to maintain periodontal health [5, 11, 49, 60].

Secondly, 1,25(OH)_2_D/VDR signalling can downregulate transcription of genes encoding proinflammatory cytokines, can suppress cyclo-oxygenase-2 (COX-2) and prostaglandin pathways, and can inhibit production of matrix metalloproteinases. Taken together, all these effects of 1,25 dihydroxyvitamin D/VDR signalling may reduce the bacteria-induced inflammatory process of periodontal disease [49, 55, 60].

Thirdly, 1,25(OH)_2_D/VDR signalling plays some supportive role in wound healing. It mediates proliferation and differentiation of keratinocytes and recruitment of monocytes/macrophages during the inflammatory phase of tissue repair, and VDR-deficient laboratory animals show impairment in the formation of granulation tissue characterized by low vascularization and extracellular matrix content [61]. Thus, vitamin D deficiency may inhibit periodontal tissue healing.

Lastly, in response to bacterial stimulation, 1,25(OH)_2_D/VDR signalling in activated keratinocytes, monocytes, and macrophages of the periodontium, may induce the production of the antibacterial agents cathelicin and *β*-defensin [5, 9, 11, 49], thus playing a role in reducing the bacterial burden; and supplementation with vitamin D may amplify the local antimicrobial effector response against periodontopathic bacteria [5, 49].

## 5. Comments

It is difficult to interpret the information from observational cross-sectional clinical studies of the association between the incidence and severity of chronic periodontitis and vitamin D serum levels [6, 14–17, 62]. Follow-up periods have been short, study populations often heterogeneous, and risk factors such as ethnicity, old age, and smoking are common to both subjects with low-levels serum vitamin D and subjects with chronic periodontitis [6, 13–15, 17, 62]. Furthermore, the effects of supplementation of calcium together with vitamin D was investigated in some of the studies, thus ruling out any conclusion as to the effect of vitamin D alone [63].

Epidemiologically, the extent, severity, and rate of progression of chronic periodontitis are extremely variable [63, 64], and as the tissue destruction in chronic periodontitis is known to be episodic with short periods of disease activity and longer periods of disease quiescence, follow-up periods of several years would be necessary to detect whether vitamin D supplementation could reduce the risk and the severity of chronic periodontitis [63].

In general, observational cross-sectional studies, like most of those investigating the link between vitamin D and chronic periodontitis, provide only weak evidence of any causal association, and as such are of questionable value in determining whether or not vitamin D deficiency increases the risk of chronic periodontitis [5, 65]. Either longitudinal cohort studies, case controlled studies, or randomized controlled trials would be needed to demonstrate any causal associations or to determine the value of vitamin D in the maintenance of periodontal health or in periodontal treatment [65].

In any case, as medicine is not a precise science, treatment modalities which according to evidence-based research have not been found to be completely effective for an observed group may still be beneficial for a subset of the general population [66]. Therefore, the use of vitamin D supplementation as an adjuvant to standard periodontal treatment should be considered in subjects with vitamin D deficiency.

## 6. Conclusion

Studies investigating the link between vitamin D and chronic periodontitis do not provide direct experimental evidence of any causal association between vitamin D deficiency and chronic periodontitis, or that vitamin D supplementation has any beneficial role in the treatment of chronic periodontitis; these studies do provide coherent and consistent evidence of the potential role of vitamin D in maintaining oral health. However, there appears to be no justification for vitamin D screening for persons with chronic periodontitis who are not at risk of vitamin D deficiency.
